# The effects of background music on neural responses during reading comprehension

**DOI:** 10.1038/s41598-020-75623-3

**Published:** 2020-10-29

**Authors:** Meng Du, Jun Jiang, Zhemin Li, Dongrui Man, Cunmei Jiang

**Affiliations:** 1 Department of Psychology, Shanghai Normal University, Shanghai, 200234 China; 2 Music College, Shanghai Normal University, Shanghai, 200234 China

**Keywords:** Human behaviour, Language, Reading

## Abstract

The effects of background speech or noise on visually based cognitive tasks has been widely investigated; however, little is known about how the brain works during such cognitive tasks when music, having a powerful function of evoking emotions, is used as the background sound. The present study used event-related potentials to examine the effects of background music on neural responses during reading comprehension and their modulation by musical arousal. Thirty-nine postgraduates judged the correctness of sentences about world knowledge without or with background music (high-arousal music and low-arousal music). The participants’ arousal levels were reported during the experiment. The results showed that the N400 effect, elicited by world knowledge violations versus correct controls, was significantly smaller for silence than those for high- and low-arousal music backgrounds, with no significant difference between the two musical backgrounds. This outcome might have occurred because the arousal levels of the participants were not affected by the high- and low-arousal music throughout the experiment. These findings suggest that background music affects neural responses during reading comprehension by increasing the difficulty of semantic integration, and thus extend the irrelevant sound effect to suggest that the neural processing of visually based cognitive tasks can also be affected by music.

## Introduction

The human brain usually must manage multi-modal information, such as visual and auditory information, simultaneously in the real world. Auditory inputs can hinder visual processing when both visual and auditory stimuli are presented^1–3^. Even if people are instructed to focus on visual inputs while ignoring auditory inputs, the ignored background sounds still interfere with visual processing. A typical example of this phenomenon is the irrelevant speech effect (for a review, see Vasilev et al.^4^), suggesting that task-irrelevant background speech disrupts the recall of visually presented digits^5,6^ and text^7,8^, proofreading^9^, and sentence or passage comprehension^10–12^. This effect could be attributed to the same cognitive process used for focal tasks, such as semantic processing, when meaningful speech is used as the background stimulus^13,14^. However, even when the background sounds are noise, this interference effect also occurs^15–17^. Such an effect might be explained by the limited capacity theory of Kahneman^18^, which posits that the amount of attention is limited, and performing multiple tasks leads to a competition for limited resources when their combined demands exceed the available resources, resulting in poor performance on one task due to an insufficient supply of attention.

Unlike speech or noise, music has a remarkable function of evoking and affecting listeners’ emotions^19,20^. Background music provides a unique window into how the brain works when music and cognitive tasks are presented simultaneously. Previous studies have primarily focused on the effects of background music on reading comprehension. Some behavioural studies have shown that reading comprehension can be improved using background music, such as Mozart’s music^21–23^, highly repetitive music with a narrow tonal range^24^ and songs^25^. In contrast, other studies have shown negative effects of background music on reading comprehension using hip-hop music^26^, UK garage-style music^17^, slow-tempo music by Bach^27^, fast and loud music^28^, familiar non-lyrical music^29,30^, and songs^31–34^ as background music. The discrepancy between these behavioural studies could be due to the differences in music and listeners. Indeed, the effects of background music on reading comprehension depend on music style^26^, music characteristics (such as tempo and complexity)^24,27^, and lyrics^32^. On the other hand, some individual factors, such as musical preferences^35^ and music expertise^36^, have been suggested to influence the effects of background music. For example, non-preferred, rather than preferred, background music disrupts reading comprehension^35^. Similarly, background music interferes with reading comprehension for musicians but not for non-musicians^36^.

Notably, the aforementioned findings were drawn from behavioural investigations. To our knowledge, only one EEG study has examined the effects of the *type* of background music on cognitive performance, brain wave activity, and heart rate during reading comprehension^37^. In that study, classical and dubstep music pieces were used as background music. Although the reading comprehension performance was better with the classical music than with the dubstep music background at the behavioural level, the *type* of background music had no effect on brain activity or physiological responses during reading comprehension. Indeed, even during face encoding, no differences in cortical activity between the background music and silence conditions were found^38^. The absence of the effect of background music is consistent with a recent study suggesting that background music has no effect on inhibitory functions, as evidenced by no differences in influences on inhibitory functions among relaxing, exciting background music and silence conditions at both the behavioural and electrophysiological levels^39^. To date, however, little is known about how the brain works when reading tasks are accompanied by background music or not. Thus, one goal of the present study was to investigate how background music affects neural responses during reading comprehension using ERPs.

When music and cognitive tasks are presented successively, music listening can induce a positive mood, increase arousal levels, and improve subsequent cognitive processing^40^. Indeed, this facilitatory effect has been confirmed in spatial tasks^40–43^. Because the music and cognitive task were presented successively in these studies, further examining whether musical arousal can affect cognitive processing when music and cognitive tasks are presented simultaneously is important. Thus, the second goal of the present study was to investigate whether the arousal level of background music modulates the neural responses during reading comprehension.

The present study focused on world knowledge, an essential component of reading comprehension^44^. Indeed, successful reading comprehension and language understanding have been suggested to rely on the utilization of acquired world knowledge^45,46^. Previous studies have demonstrated that world knowledge violations elicited a larger N400 than correct sentences^47–50^. The N400 is an ERP index of semantic processing (for a review, see Kutas and Federmeier^51^). The increased amplitude of N400 reflects the increased difficulty of semantic integration^52–54^, suggesting that more effort is required to integrate the meaning of a stimulus into the preceding contexts^55–57^ or prior world knowledge (for a review, see Lau, Phillips and Poeppel^58^).

Thus, the goals of the present study were to examine the effects of background music on neural responses to world knowledge integration and its modulation by musical arousal, with a 3 (group: high-arousal music, low-arousal music, and silence) × 2 (sentence type: correct vs world knowledge violation) mixed design. First, we included high- and low-arousal music as two types of background music because music-evoked arousal may mediate the effects of prior exposure to music on subsequent cognitive processing^40,59,60^. Participants reported their arousal levels during the entire experiment to demonstrate the effect of musical listening on the level of arousal. Second, each participant completed reading comprehension in one of three backgrounds, silence or low- or high-arousal music, to exclude the carry-over effect. Third, each sentence was presented word by word, and the last word of each correct sentence was changed to form a sentence with a world knowledge violation. Fourth, both the high- and low-arousal musical excerpts used in our study were unfamiliar instrumental music expressing positive emotions to control for the influences of musical familiarity on reading comprehension. Finally, three pretests were conducted to ensure the validity of the stimuli. The first pretest assessed the emotional valence and arousal levels of the background music that we used. The second pretest ensured that the originally created sentences were unambiguous, and the last pretest confirmed a significant difference in reasonableness between the two types of sentences. We expected that, if background music is as distracting as irrelevant speech^61^ or noise^17^, the N400 effect for silence should be smaller than that for background music.

## Results

### Behavioural results

The results of the mean accuracy and mean reaction times (RTs) are summarized in Table 1. Based on previous studies^62–64^, trials with incorrect judgement and trials with RTs shorter than 200 ms or longer than 1500 ms were excluded from the calculations of mean RTs. Regarding the mean accuracy, a nonparametric ANOVA-type statistic (ATS) taking group (high-arousal music, low-arousal music and silence) as the whole-plot factor and sentence type (correct vs. world knowledge violation) as the sub-plot factor was conducted. No significant effects were found for either the main effects of group [ATS(1.99) = 0.38, *p* = 0.683] and sentence type [ATS(1) = 0.99, *p* = 0.319] or the interaction between group and sentence type [ATS(1.86) = 0.92, *p* = 0.392]. For the mean RTs, a two-way mixed analysis of variance (ANOVA) taking group as the between-subjects factor and sentence type as the within-subjects factor revealed no significant effects for either the main effects for group [*F*(2, 36) = 1.65, *p* = 0.207, η_p_^2^ = 0.08] and sentence type [*F*(1, 36) = 1.53, *p* = 0.224, η_p_^2^ = 0.04] or their interaction [*F*(2, 36) = 0.66, *p* = 0.525, η_p_^2^ = 0.04]. These results suggested that our participants concentrated on the reading task during the experiment and understood the sentences well.

**Table 1:** Mean accuracy and RTs of correct sentences and sentences with world knowledge violations for the three groups.

Group	Correct		World knowledge violation	
	*M*	*SD*	*M*	*SD*
**Accuracy (%)**				
Silence	96.00	2.38	96.62	3.20
Low-arousal music	92.31	9.80	94.38	7.57
High-arousal music	95.08	4.29	94.92	4.17
**RTs (ms)**				
Silence	788.16	135.78	786.84	158.32
Low-arousal music	863.64	147.65	888.35	169.30
High-arousal music	793.81	113.93	804.86	85.64

**Figure 1:**
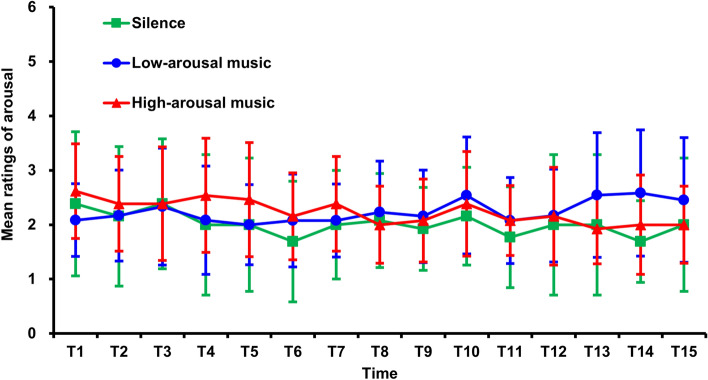
Mean arousal ratings over fifteen time points for the three groups. The error bars indicate the standard deviations.

To examine whether background music can induce emotional arousal, the participants’ arousal levels were reported 15 times throughout the experiment. A nonparametric ATS taking group as the whole-plot factor and time (T1, T2, T3, …, T15) as the sub-plot factor was conducted. As shown in Fig. 1, neither the main effects for group [ATS(1.93) = 0.68, *p* = 0.502] and time [ATS(7.02) = 1.44, *p* = 0.183] nor their interaction was significant [ATS(10.61) = 1.14, *p* = 0.329]. These results indicated that the participants’ arousal levels were not affected by the background music.

### Electrophysiological results

**Figure 2:**
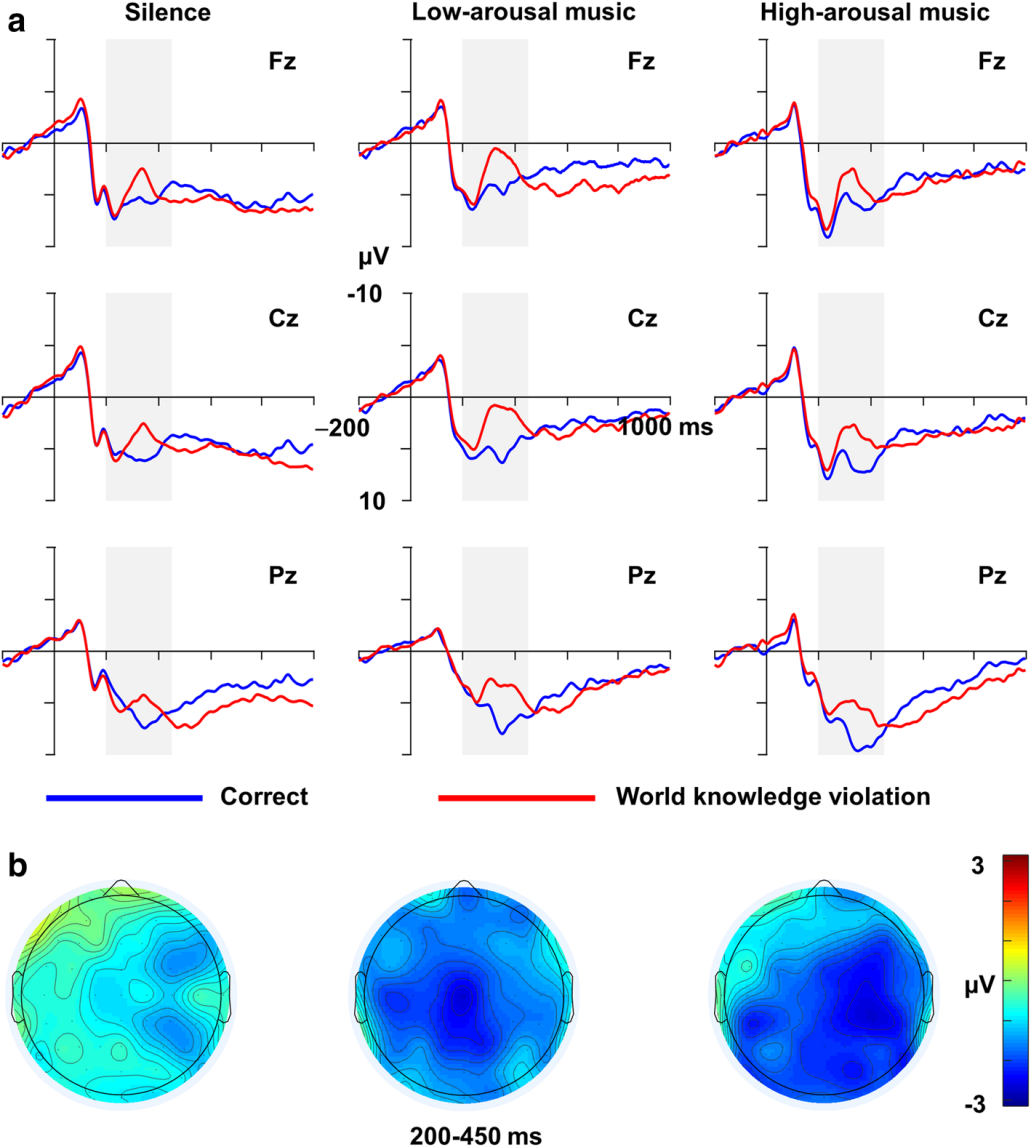
Grand mean ERPs for the three groups at the selected scalp sites as a function of sentence type (**a**). Grey-shaded areas indicate the time window used for statistical analysis. Scalp topographies of the N400 (200–450 ms) for each group (**b**).

Figure 2 shows the electric brain responses to correct sentences and sentences with world knowledge violations and topographical maps for the different groups. As shown, world knowledge violations elicited a larger N400 than correct sentences in the time windows of 200–450 ms, with a broad scalp distribution. However, the magnitude of the N400 effect seemed to differ between the groups with and without background music. Because we focused on the influences of background music and the differences associated with the N400 effect, only the significant effects related to group or sentence type are reported in the following paragraphs.

For the midline electrodes, a three-way mixed ANOVA taking group as the between-subjects factor and sentence type and anteriority (anterior, central and posterior) as the within-subjects factors was conducted. A significant main effect was found for sentence type [*F*(1,36) = 118.36, *p* < 0.001, η_p_^2^ = 0.77], indicating that world knowledge violations elicited a larger N400 than correct sentences. A significant two-way interaction was also found between group and sentence type [*F*(2,36) = 9.22, *p* = 0.001, η_p_^2^ = 0.34] owing to a larger N400 elicited by world knowledge violations than by correct sentences in the silence [*F*(1,36) = 8.03, *p* = 0.008, η_p_^2^ = 0.18], low-arousal music [*F*(1,36) = 73.17, *p* < 0.001, η_p_^2^ = 0.67], and high-arousal music groups [*F*(1,36) = 55.61, *p* < 0.001, η_p_^2^ = 0.61]. Furthermore, a significant two-way interaction between sentence type and anteriority was also observed [*F*(1.59,57.15) = 8.61, *p* = 0.001, η_p_^2^ = 0.19], reflecting that a larger N400 was elicited by world knowledge violations than by correct sentences in the anterior [*F*(1,36) = 22.13, *p* < 0.001, η_p_^2^ = 0.38], central [*F*(1,36) = 113.24, *p* < 0.001, η_p_^2^ = 0.76], and posterior regions [*F*(1,36) = 116.76, *p* < 0.001, η_p_^2^ = 0.76].

A two-way mixed ANOVA taking group as the between-subjects factor and anteriority as the within-subjects factor was conducted to further examine whether differences existed in the magnitude of the N400 effect among the three groups. A significant main effect was found for group [*F*(2,36) = 9.20, *p* = 0.001, η_p_^2^ = 0.34]. Pairwise comparisons revealed a smaller N400 effect in the silence group than in the low-arousal music (*p* = 0.001) and high-arousal music groups (*p* = 0.007), but the latter two groups did not differ from each other (*p* > 0.05). A significant main effect was also found for anteriority [*F*(1.43,51.29) = 8.96, *p* = 0.002, η_p_^2^ = 0.20]. Pairwise comparisons showed a larger N400 effect in the central versus anterior region (*p* < 0.001), but no differences were found between the central and posterior regions (*p* > 0.05) or between the anterior and posterior regions (*p* > 0.05). The interaction between group and anteriority was not significant (*p* > 0.05).

For the electrodes in the lateral regions, a four-way mixed ANOVA taking group as the between-subjects factor and sentence type, anteriority and hemisphere (left vs. right) as the within-subjects factors was conducted. A significant main effect of sentence type was found [*F*(1,36) = 113.29, *p* < 0.001, η_p_^2^ = 0.76], indicating that world knowledge violations elicited a larger N400 than correct sentences. A significant two-way interaction was also found between group and sentence type [*F*(2,36) = 7.13, *p* = 0.002, η_p_^2^ = 0.28] owing to a larger N400 elicited by world knowledge violations than by correct sentences in the silence [*F*(1,36) = 9.64, *p* = 0.004, η_p_^2^ = 0.21], low-arousal music [*F*(1,36) = 52.15, *p* < 0.001, η_p_^2^ = 0.59], and high-arousal music groups [*F*(1,36) = 65.75, *p* < 0.001, η_p_^2^ = 0.65]. A significant two-way interaction between group and hemisphere was also observed [*F*(2,36) = 3.36, *p* = 0.046, η_p_^2^ = 0.16], indicating that both the low-arousal music [*F*(1,36) = 6.82, *p* = 0.013, η_p_^2^ = 0.16] and high-arousal music groups [*F*(1,36) = 29.18, *p* < 0.001, η_p_^2^ = 0.45] exhibited stronger neural responses in the left than right hemisphere, while the silence group showed a marginally significant difference in neural responses between the left and right hemispheres [*F*(1,36) = 3.80, *p* = 0.059, η_p_^2^ = 0.10]. Furthermore, the two-way interaction between sentence type and anteriority was also significant [*F*(1.33,48.00) = 4.50, *p* = 0.029, η_p_^2^ = 0.11], indicating that a larger N400 was elicited by world knowledge violations than by correct sentences in the anterior [*F*(1,36) = 26.82, *p* < 0.001, η_p_^2^ = 0.43], central [*F*(1,36) = 109.78, *p* < 0.001, η_p_^2^ = 0.75], and posterior regions [*F*(1,36) = 156.93, *p* < 0.001, η_p_^2^ = 0.81].

A nonparametric ATS taking group as the whole-plot factor and anteriority and hemisphere as the sub-plot factors was conducted to further examine whether differences existed in the magnitude of the N400 effect among the three groups. A significant main effect was found for group [ATS(1.94) = 5.55, *p* = 0.004]. Pairwise comparisons revealed a smaller N400 effect in the silence group than in the low-arousal music (*p* = 0.018) and high-arousal music groups (*p* = 0.012), but the latter two groups did not differ from each other (*p* > 0.05). A significant main effect was also found for anteriority [ATS(1.27) = 4.37, *p* = 0.027]. Pairwise comparisons revealed a larger N400 effect in the central than in the anterior region (*p* < 0.001), but no differences were found between the central and posterior regions (*p* > 0.05) or between the anterior and posterior regions (*p* > 0.05). No other main effects or interactions were significant (*ps* > 0.05).

## Discussion

The present study used ERPs to investigate the effects of background music on neural responses during reading comprehension and its modulation by musical arousal level. The results showed that a larger N400 was elicited in response to world knowledge violations than correct controls during reading comprehension either with or without background music. However, the N400 effect for silence was significantly smaller than those for high- and low-arousal music backgrounds, with no significant difference between the two musical backgrounds. The arousal levels of the participants were not affected by the high- and low-arousal music during the experiment. These findings suggest that background music influenced the neural responses during reading comprehension, and the musical arousal level did not alter the effects of background music on reading comprehension.

The main finding of the present study is that reading comprehension elicited a larger N400 effect with background music than without background music. The classical N400 effect, which manifests in a larger negative amplitude for semantically incongruent sentences than for congruent sentences, reflects semantic processing^51,65,66^. This N400 effect has also been observed in response to sentences with world knowledge violations^47–50,67–69^. The amplitude of N400 is assumed to reflect the difficulty of integrating the coming word into the preceding context^52–54^. The higher that the difficulty of integrating the violations into the preceding context or world knowledge is, and the greater that the efforts deployed by the brain for the integration are, the larger that the N400 is^52–57^. Therefore, the different N400 effects in our study could indicate that the background music groups required more effort deployed by the brain to integrate violated words into pre-existing world knowledge than the silence group. In other words, compared with the silent context, the presence of background music increased the difficulty of neural processing during reading comprehension.

Our findings can be interpreted according to the limited capacity theory^18^ and the distraction hypothesis^70^, suggesting that individuals’ attention resources are limited and that concurrent tasks compete for available attention. When the required resources exceed the available resources, the tasks interfere with each other. In the present study, because the presence of background music might demand attention resources, the attention resources used to complete reading comprehension were reduced, resulting in difficulties in sentence integration, eventually manifested as a larger N400 effect.

Another finding of the present study is that no significant difference was observed in the N400 effect between the high- and low-arousal music groups, consistent with Burkhard et al.^39^ who showed no different effects on inhibitory function between the relaxing and exciting background music conditions. This finding could be attributed to the constant arousal levels of our participants during the entire experiment. Specifically, neither the high- nor low-arousal background music induced emotional arousal during the experiment. Indeed, previous studies have also found that background music fails to induce emotional arousal^71,72^. The failure to induce emotional arousal during cognitive processing could be explained by the characteristics of the background music. Emotionally touching background music can possibly enhance participants’ arousal levels relative to background music that is not emotionally touching^73^. On the other hand, the competition for attention resources during cognitive processing could also account for the failure to induce emotional arousal. Specifically, although background music affected reading comprehension in the present study, the attention resources available for listening to background music were limited due to competition for attention resources. In this case, background music might not be sufficient to increase participants’ arousal levels when presented with reading tasks.

Although the reading stimuli in the present study were written in Chinese, an ideographic language, this fact is not a limitation of the study. Specifically, previous studies have demonstrated that world knowledge violations can elicit an N400 effect relative to correct controls, not only in Chinese^48,68^ but also in other languages using alphabets, such as English^49,69^, Dutch^50,74^ and German^47,67,75^. These findings indicate that the difference between ideographic and alphabetic languages does not affect the neural processing of world knowledge integration in sentence comprehension. On the other hand, regarding the background music, our background stimuli were selected from Western tonal music composed in the Baroque and Classical periods. It is well known that Western tonal music has been widely spread in many areas of the world. Due to familiarity with tonal conventions of Western music, both Western^76,77^ and Chinese listeners^78,79^ can process Western tonal structures and exhibit similar neural responses to these tonal structures. Therefore, our findings could be applicable to many other populations who speak alphabetic languages.

In conclusion, the present findings showed that the presence of background music influences neural responses during reading comprehension regardless of whether the music is of high or low arousal. Our findings extend the irrelevant sound effect, suggesting that the processing of visually based cognitive tasks can be disrupted not only by task-irrelevant background speech^6,7,9,10^ or noise^15–17^ but also by music. Indeed, when music is presented prior to the cognitive task, a unique facilitatory effect of music occurs on non-music cognitive processing because prior exposure to music can induce participants’ emotions and subsequently improve subsequent cognitive tasks^40,59,60^. However, when presented simultaneously with the reading task, neither high- nor low-arousal music increased participants' arousal levels. In this case, background music might become a source of distraction for reading tasks since both compete for available attention, thus increasing the difficulty of semantic integration during reading comprehension.

## Methods

### Participants

A prior power analysis using G*Power software, version 3.1.9.4^80^, was conducted to determine the minimum sample size. To detect interactions with 80% statistical power, an alpha level of 0.05, and a medium effect size (ƒ = 0.25), we needed at least 12 participants in each group. Given that the habit of using background music can affect reading comprehension^25,34,81^, 39 postgraduates who preferred listening to music (n = 26) or a silent environment (n = 13) during reading were recruited for this study to control for the potential effect of this habit. They were then assigned to the low- and high-arousal music groups or the silence group based on their reading habits. The three groups (13 participants for each group) were matched by sex, age, and years of education (see Table 2). All of the participants were right-handed, with normal hearing and normal or corrected-to-normal vision. None had received musical training, and had any previous history of psychiatric or neurological disorders. The protocol for the experiment was approved by the Ethics Committee of Shanghai Normal University in China and conducted in line with the Declaration of Helsinki. All of the participants provided informed consent prior to the experiment and were paid for their participation.

**Table 2:** Demographic information of the participants.

Variable	Group			Statistical value	*p*	η_p_^2^
	Silence	Low-arousal music	High-arousal music			
Age	24.38 (1.12)	24.46 (1.13)	25.15 (1.72)	*F*(2, 38) = 1.27	0.292	0.05
Sex (male:female)	5:8	5:8	5:8	*χ*^2^(2) = 0.00	1.000	0.00
Years of education	16.46 (0.66)	16.62 (0.77)	16.77 (0.93)	*F*(2, 38) = 0.49	0.617	0.03

Standard deviation values are shown in parentheses.

### Stimuli

For background music stimuli, six pieces of fast-tempo and six pieces of slow-tempo music in major mode were originally selected as the high- and low-arousal music clips, respectively, given that major mode and fast-tempo music tends to induce a positive mood and increase arousal levels, whereas minor mode and slow-tempo music tends to induce a more negative mood and lower arousal levels^59^. All music excerpts were orchestral music without voice or lyrics selected from Western tonal music composed in the Baroque, Classical, or Romantic periods. The music stimuli were normalized to − 3 dB and saved as monaural .wav files with a sampling rate of 44.1 kHz and 16-bit resolution by means of Adobe Audition software, version CS6 (Adobe System Inc., San Jose, CA, USA).

A pretest was conducted to assess the emotional valence and arousal levels of the selected music excerpts. Sixteen musically untrained participants who preferred listening to music during reading were asked to rate each music excerpt with regard to perceived valence and arousal on two 6-point scales (valence: 1 = *very negative*, 6 = *very positive*; arousal: 1 = *very calming*, 6 = *very exciting*). Moreover, they were asked to report whether they were familiar with the music excerpts. None of them participated in the subsequent ERP experiment. To avoid distraction from the reading task resulting from changes in different music excerpts during playing, only two unfamiliar music excerpts with the highest or lowest arousal level were chosen as the background music stimuli. Specifically, the high-arousal music excerpt was selected from Handel’s Oboe Sonata in B-flat Major, HWV 357, Movement I (Andante), while the low-arousal music was selected from Mozart’s Violin Concerto No.1 in B-flat Major, K.207, Movement III (Presto). Paired sample *t* tests showed that high-arousal (arousal: *M* = 5.44, *SD* = 0.63; valence: *M* = 5.13, *SD* = 0.81) and low-arousal music (arousal: *M* = 3.38, *SD* = 0.81; valence: *M* = 4.75, *SD* = 0.68) differed significantly in perceived arousal levels [*t*(15) =  − 10.69, *p* < 0.001, *d* = 2.80] and matched in perceived valence [*t*(15) =  − 1.70, *p* = 0.111, *d* = 0.50].

For sentence stimuli, 90 original Chinese sentences expressing world knowledge were created. Each sentence consisted of three to seven words. The second pretest was conducted to ensure that all of the sentences were unambiguous. Nine participants who did not participate in the formal experiment read each sentence in which the last word (critical word) had been deleted, and then completed the sentence with the word that they thought was most reasonable. Thus, 77 sentences to which all of the participants answered correctly were chosen as correct sentences. Seventy-seven sentences with world knowledge violations were then created by replacing the last word of the correct sentences with a word that violated world knowledge (see Table 3). The word frequency of the last word in the two types of sentences was matched (*p* > 0.05). A third pretest was conducted to determine whether a difference existed in reasonableness between the two types of sentences. Twelve participants not participating in the formal experiment were recruited to rate the reasonableness of all 154 sentences on a 5-point scale (from 1 = *very unreasonable* to 5 = *very reasonable*). Paired sample *t*-tests showed that correct sentences (*M* = 4.69, *SD* = 0.25) and sentences with world knowledge violations (*M* = 1.06, *SD* = 0.05) differed significantly in reasonableness (*t*(11) = 53.28, *p* < 0.001, *d* = 15.84).

**Table 3:** Example sentences of reading comprehension materials with English translations.

Sentence type	Chinese sentence	English translations
Correct sentence	划船需要用桨。	Rowing requires oars
	姚明擅长打篮球。	Yao Ming is good at playing basketball
	美国的现任总统是特朗普。	The current President of the US is Trump
Sentence with world knowledge violation	划船需要用布。	Rowing requires cloth
	姚明擅长打排球。	Yao Ming is good at playing volleyball
	美国的现任总统是华盛顿。	The current President of the US is Washington

The critical words are underlined.

### Procedures

**Figure 3:**
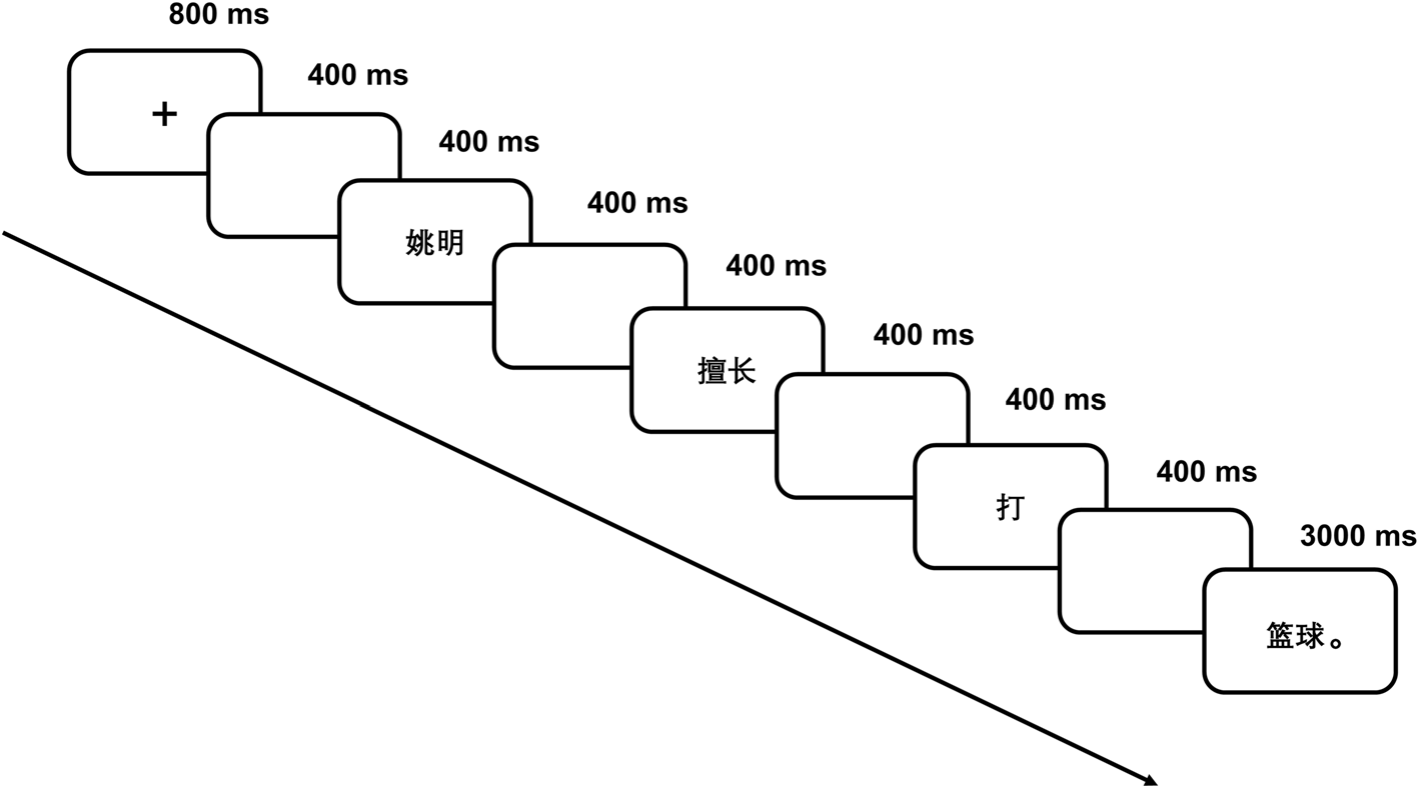
Trial scheme with detailed time sequence for the experiment.

Stimulus presentation and response timing were controlled by E-Prime 1.0 (Psychology Software Tools Inc., Sharpsburg, PA, USA), on a computer. Before the formal experiment, four trials were administered for practice. Each trial started with a red fixation point in the middle of the screen with a black background for 800 ms, followed by a 400-ms blank screen. After the blank screen, a sentence was presented word by word. The duration of each word presentation was 400 ms, except that the last critical word with a dot was presented for 3000 ms. A 400-ms blank screen appeared between subsequent words. To maintain participants’ attention on the reading comprehension task, when the critical word appeared, the participants were instructed to press either the F key with the left hand or the J key with the right hand on a standard keyboard to indicate whether the sentence was correct. The association between response button (F or J) and response (correct or incorrect) was counterbalanced across participants in each group. Given that the dominant hand responds more rapidly than the non-dominant hand in motor tasks^82,83^, counterbalancing between the response button and response would control for the interference effects of handedness. Additionally, the counterbalancing design might avoid any lateralization of topographies associated with particular response button assignments. The trials were presented in pseudorandom order such that the same sentence type was maximally presented three times in a row. The trial scheme with detailed time sequence is shown in Fig. 3.

During the trials, background music was played throughout via Edifier R101V loudspeakers (Edifier Technology Co., Ltd., Beijing, China) for the high- and low-arousal music groups, while no music was played for the silence group. EEG recording started after practice trials and ended after completing the task. In addition, the arousal levels experienced by the participants were measured using a 6-point scale (from 1 = *very calming* to 6 = *very exciting*). Throughout the experiment, a total of 15 arousal ratings were obtained from each participant. To control for familiarity with the music and musical preference, following the ERP experiment, the participants were asked to report whether they had heard the music before and whether they liked it. All of the participants reported being unfamiliar with the music and liking it.

### EEG recording and data analysis

EEG activity was continuously recorded from 64 Ag/AgCl scalp electrodes positioned on an elastic cap according to the international 10–20 system using the ActiveTwo Biosemi System (Biosemi, Amsterdam, Netherlands). The Common Mode Sense (CMS) active electrode and the Driven Right Leg (DRL) passive electrode were used as the reference and ground, respectively. EEG signals were recorded at a sampling rate of 2048 Hz.

The acquired EEG signals were preprocessed offline using the EEGLAB 14.1.2b^84^ and ERPLAB 7.0.0 toolboxes^85^ run in MATLAB 2016a (MathWorks Inc., Natick, MA, USA). To reduce the size of the data files, raw data were downsampled to 256 Hz. Data were bandpass filtered with cutoffs of 0.1 and 25 Hz. Subsequently, data with large artefacts caused by body movements, channel drifts and muscle activity were first rejected manually. The data were then referenced to the average activity of the left and right mastoid electrodes. Epochs were extracted ranging from 200 ms before to 1000 ms after the onset of the critical word with a baseline interval from -200 to 0 ms. Next, all of the segmented data were subjected to independent component analysis (ICA) to identify components associated with eye blinks and eye movements. Individual components were inspected, and components associated with eye blinks and eye movements were removed. Additionally, using an automatic moving window peak-to-peak function with a window width of 200 ms and a step size of 100 ms, epochs were rejected as artefacts when the voltage exceeded 100 μV. Based on the behavioural data, only trials with correct responses were finally averaged by each condition for each participant at each electrode. Specifically, for the correct sentence, the mean number of valid trials was 66.77 (*SD* = 6.30) in the silence condition, 63.23 (*SD* = 11.48) in the low-arousal music condition, and 60.23 (*SD* = 8.80) in the high-arousal music condition. For sentences with world knowledge violations, the mean number of valid trials was 67.38 (*SD* = 7.76) in the silence condition, 63.15 (*SD* = 12.37) in the low-arousal music condition, and 60.85 (*SD* = 10.07) in the high-arousal music condition. A non-parametric ANOVA-type statistic showed no significant difference in the mean number of valid trials across all conditions (*ps* > 0.05).

Based on visual inspection and previous studies of language comprehension^86,87^, a time window of 200–450 ms (i.e., N400 component) after the onset of the critical word was used for statistical analysis. We computed the mean amplitude values for nine regions of interest (ROIs): left anterior (FP1, AF7, AF3, F5, F3, and F1), left central (FC5, FC3, FC1, C5, C3, C1, CP5, CP3, and CP1), left posterior (P5, P3, P1, PO3, and O1), right anterior (FP2, AF8, AF4, F6, F4, and F2), right central (FC6, FC4, FC2, C6, C4, C2, CP6, CP4, and CP2), right posterior (P6, P4, P2, PO4, and O2), anterior midline (FPz, AFz, and Fz), central midline (FCz, Cz, and CPz), and posterior midline (Pz, POz, and Oz). When the data met the assumption of normality (Shapiro–Wilk test with *p* > 0.05), mixed ANOVA was performed with SPSS 25 (IBM SPSS Inc., Chicago, IL, USA), for the electrodes in the midline and lateral regions separately. Nevertheless, when the data deviated from normality (Shapiro–Wilk test with *p* < 0.05), the nonparametric ATS was conducted with the nparLD 2.1^88^ package in R software, version 3.6.3. For the electrodes in the midline regions, group (high-arousal music, low-arousal music and silence) was considered as the between-subjects factor, whereas sentence type (correct vs. world knowledge violation) and anteriority (anterior, central and posterior) were considered as the within-subjects factors. For the electrodes in the lateral regions, hemisphere (left vs. right) was added as an additional within-subjects factor. In addition, to compare the magnitude of the N400 effect, statistical analysis was also performed for difference waves (subtracting the correct sentences from the sentences with world knowledge violations) in the midline and lateral regions separately. Only the significant effects containing the main experimental variables (group and sentence type) are reported. When any significant interactions were found, pairwise comparisons adjusted by Bonferroni correction were conducted. When the data violated the sphericity assumption, the degrees of freedom were adjusted with the Greenhouse–Geisser correction.
